# Exploring the role of riboflavin in swine well-being: a literature review

**DOI:** 10.1186/s40813-024-00399-1

**Published:** 2024-10-31

**Authors:** Yauheni Shastak, Wolf Pelletier

**Affiliations:** BASF SE, Nutrition & Health Division, 67063 Ludwigshafen am Rhein, Germany

**Keywords:** Vitamin B_2_, Swine, Performance, Deficiency, Requirement

## Abstract

Riboflavin (vitamin B_2_) is an essential B-vitamin crucial for the metabolism, development, and overall well-being of porcine species. As pig production intensifies, understanding the micronutrient needs of swine, particularly riboflavin, becomes increasingly vital. Riboflavin acts as a precursor for coenzymes involved in key redox reactions essential for energy production, growth, and immune regulation. Ariboflavinosis can disrupt metabolic functions, leading to impaired growth, reproductive issues, decreased feed efficiency, compromised immune function, ocular problems, and liver dysfunction. To ensure optimal growth and health, pig diets are consistently supplemented with riboflavin-enriched supplements. This review explores the diverse functions of riboflavin in swine metabolism, focusing on biochemical basics, metabolic pathways, riboflavin uptake and distribution, consequences of deficiency, and benefits of adequate intake. It emphasizes the need for optimized riboflavin supplementation strategies tailored to different production stages and environmental conditions. According to recommendations from four major breeding companies, the dietary riboflavin levels for swine are advised to range between 7.5 and 15 mg/kg for piglets, 3.5 to 8.0 mg/kg for finishing gilts and barrows, 4 to 10 mg/kg for gestating sows, and 5 to 10 mg/kg for lactating sows. Advances in precision nutrition, microbial production of riboflavin, and the development of functional feed additives are potential innovations to enhance swine health, growth performance, and sustainability. Comprehensive studies on the long-term effects of subclinical riboflavin deficiency and the broader health and welfare implications of supplementation are also needed. Addressing knowledge gaps and embracing future trends and innovations will be key to optimizing riboflavin supplementation and advancing the swine industry.

## Introduction

In the dynamic and continuously evolving field of swine production, optimal nutrition is essential for maximizing productivity and ensuring porcine well-being. As a key component of the global meat industry, swine contribute substantially to the world’s meat supply, meeting the growing demand for high-quality protein [1]. Projections indicate that pork will remain a significant part of global meat consumption through 2031, although its share may face competition from rising poultry production [2].

Among the complex nutritional requirements of swine, B-vitamins, a group of essential water-soluble compounds, are critical for numerous physiological processes [3]. In particular, riboflavin stands out for its vital role in maintaining metabolic balance, promoting growth, and supporting overall health in pigs [4, 5]. Vitamin B_2_ is a key precursor for coenzymes that drive redox reactions crucial for energy production, growth, and immune regulation [6, 7].

Swine have high metabolic demands due to their rapid growth rates and efficient production capabilities, necessitating substantial nutritional inputs [8, 9]. Riboflavin serves as a precursor for two essential coenzymes: flavin mononucleotide (FMN) and flavin adenine dinucleotide (FAD). These coenzymes are integral to mitochondrial electron transport and various oxidative reactions [10, 11], processes that are vital for the metabolism of carbohydrates, fats, and proteins, ultimately leading to the production of adenosine triphosphate (ATP), the primary energy carrier in cells [12, 13]. Without adequate riboflavin, metabolic functions are disrupted, which can result in growth retardation, reproductive issues, and decreased feed efficiency [5, 14].

Plants are the primary producers of vitamin B_2_, making plant-based feedstuffs the main source of riboflavin in swine diets [15, 16]. However, the riboflavin content in these plant-derived feeds can vary significantly depending on factors such as soil conditions, weather patterns, and farming practices [17]. Due to these fluctuations, it is crucial to supplement pig diets with riboflavin-enriched supplements to ensure that their nutritional needs are met, thus supporting optimal growth and health.

This review explores the diverse roles of vitamin B_2_ in pig metabolism, development, and overall well-being, focusing on several key areas. First, it covers the biochemical basics and metabolic pathways of riboflavin, followed by an examination of its uptake and distribution within the body. It then reviews riboflavin requirements and addresses the potential health impacts of riboflavin deficiency. Following this, the benefits of adequate intake are discussed, particularly in relation to performance, antioxidant capacity, and immune function. The review concludes with insights into current research and future directions. By delving into the complex functions of riboflavin in pig biology, experts in nutrition, research, and production can craft novel dietary approaches to enhance the swine sector in a sustainable manner.

## Biochemical basics and metabolic pathways

**Fig. 1:**
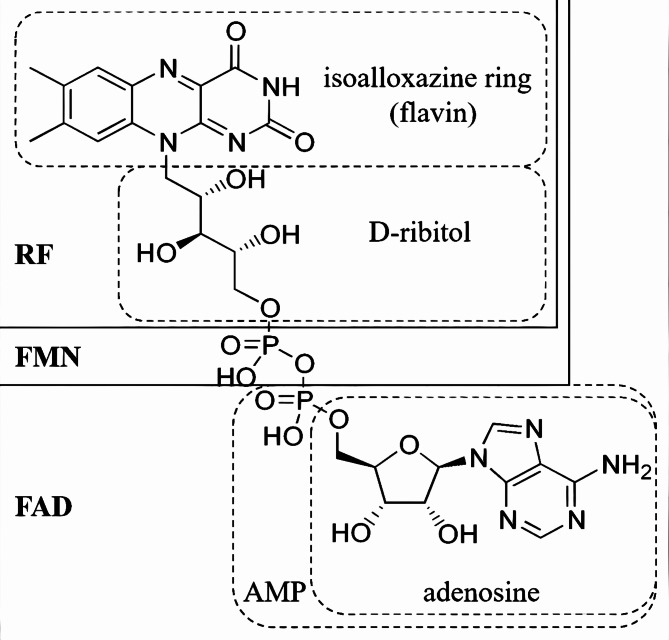
Chemical structure and nomenclature of flavins [20]. RF = riboflavin; FMN = flavin mononucleotide; FAD = adenine dinucleotide; AMP = adenosine monophosphate

Structurally, riboflavin features a unique heterocyclic ring system, combining an isoalloxazine ring with a ribitol side chain [7]. This configuration enables riboflavin to be converted into FMN and FAD, two coenzymes that play crucial roles in various enzymatic reactions (Fig. 1). FMN is formed by the phosphorylation of riboflavin, catalyzed by riboflavin kinase in the presence of ATP, and is further converted into FAD by FAD synthetase [18, 19].

Riboflavin’s biochemical activities in pigs are intricately linked to its function as a cofactor in oxidation-reduction processes. FMN and FAD participate in various critical metabolic pathways, notably the electron transport chain, where they assist in electron transfer and are crucial for oxidative phosphorylation [21, 22]. Specifically, FAD-containing NAD(P) transhydrogenases help pump protons across the inner mitochondrial membrane, which is linked to ATP production. In the glycerol phosphate shuttle, the mitochondrial enzyme glycerol 3-phosphate dehydrogenase (which contains FAD) works with a cytoplasmic glycerol 3-phosphate dehydrogenase (which does not contain FAD) [23].

The metabolism of several vitamins involves flavoproteins, a group of proteins that contain a nucleic acid derivative of riboflavin as a prosthetic group. For example, riboflavin is crucial for the conversion of tryptophan to niacin, another vital B-vitamin, with the flavoprotein kynurenine 3-monooxygenase serving as a key enzyme in this metabolic process [23, 24]. The intermediates and metabolites produced in the kynurenine pathway eventually lead to the formation of quinolinic acid, a precursor for NAD + synthesis, an essential coenzyme and the active form of vitamin B_3_ (niacin) involved in many cellular processes, including energy metabolism, DNA repair, and cell signaling [25–28].

The FAD-dependent enzyme methylene tetrahydrofolate reductase (MTHFR) is essential for folate (vitamin B_9_) metabolite recycling [29]. MTHFR catalyzes the conversion of 5,10-methylene tetrahydrofolate to 5-methyl tetrahydrofolate [30]. Reduced activity of MTHFR necessitates a higher intake of folate to prevent deficiency.

Riboflavin is also involved in hormone metabolism. Monoamine oxidase, which contains FAD, is the primary enzyme responsible for breaking down amine neurotransmitters such as norepinephrine, epinephrine, serotonin, and dopamine [29]. Moreover, xanthine dehydrogenase, which contains FAD, molybdopterin, and iron-sulfur clusters, is a molybdenum-containing hydroxylase involved in purine metabolism, specifically catalyzing the oxidation of hypoxanthine to xanthine and then to uric acid [30–32].

The metabolism of vitamin B_12_ involves three flavoenzymes: cob(II)alamin reductase, aquacobalamin reductase/NADPH, and aquacobalamin reductase/NADH [23]. These enzymes ensure the proper reduction and activation of cobalamin, enabling its critical roles in cellular metabolism and maintaining overall health. Another FAD-dependent enzyme, retinal dehydrogenase, catalyzes the irreversible conversion of retinal into all-trans-retinoic acid, the most biologically active form of vitamin A [31–34].

Flavoproteins are involved in enzyme complexes that facilitate the breakdown of amino acids such as glycine, glutamate, valine, leucine, and isoleucine. Examples of these enzymes include glutaryl-CoA dehydrogenase, 2-methylacyl-CoA dehydrogenase (which is involved in branched-chain amino acid catabolism), N-methyl-L-amino-acid oxidase, kynurenine 3-monooxygenase, methionine synthase reductase (which contains FAD, FMN, and cobalamin), L-amino acid oxidase, (S)-2-hydroxy-acid oxidase (which also acts as an L-amino acid oxidase and is found in peroxisomes), D-aspartate oxidase, and D-amino acid oxidase [23].

**Fig. 2:**
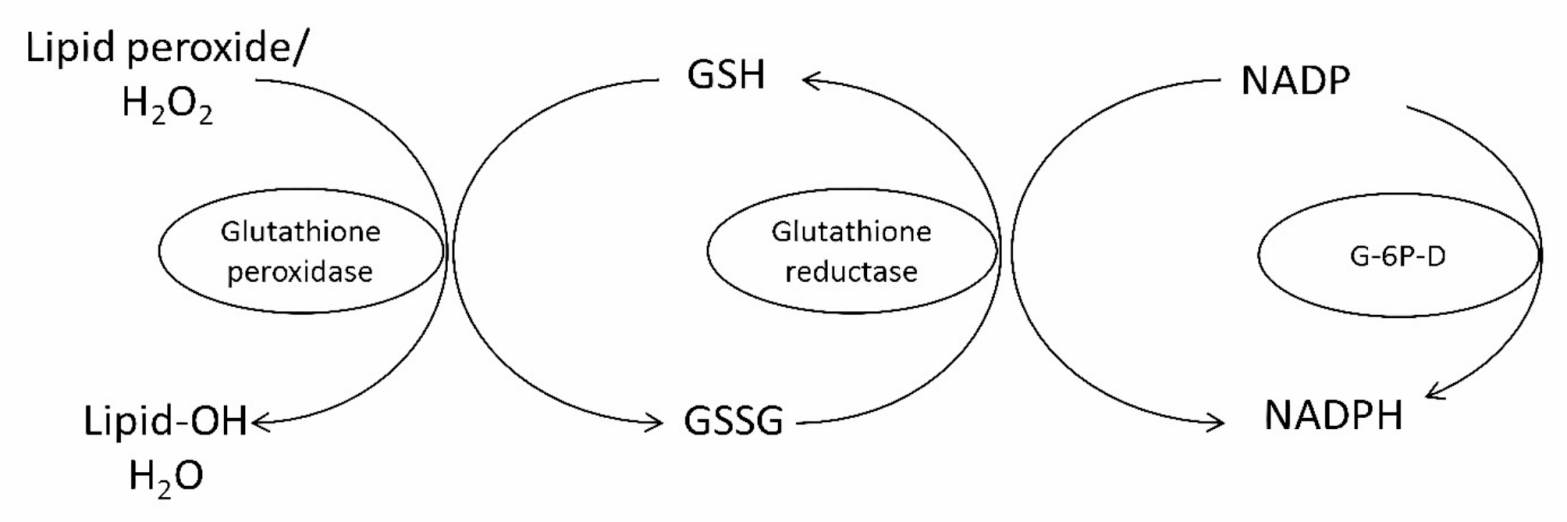
Conversion of oxidized glutathione (GSSG) to the reduced form (GSH) by glutathione reductase requires riboflavin in the flavin adenine dinucleotide (FAD) coenzyme form for its activity [7]. G-6P-D = glucose-6-phosphate dehydrogenase

Numerous flavoproteins play a crucial role in sustaining the intracellular redox balance and safeguarding sulfur-containing compounds from oxidation [7, 13, 23]. FAD-containing glutathione reductase is an enzyme that converts oxidized glutathione (GSSG) back into its reduced form (GSH) using NADPH [35, 36]. This conversion is crucial for maintaining a high ratio of GSH to GSSG, which is essential for protecting cells from oxidative stress. By ensuring sufficient levels of reduced glutathione, glutathione reductase helps sustain the cell’s antioxidant defense mechanisms (Fig. 2).

### Riboflavin uptake, distribution and functions

In swine nutrition, understanding the uptake and distribution of riboflavin is fundamental to optimizing health and growth performance. This section delves into the complex processes involved in the absorption, transportation, and tissue distribution of riboflavin.

Swine diets commonly include grains and protein-rich meals, which have varying levels of vitamin B_2_ [13] (Table 1). The majority of riboflavin in feed materials is present as free coenzymes, mainly FMN and FAD, with FAD being the most abundant [37]. Due to the natural variability in vitamin B_2_ levels in plant-based ingredients, along with factors that affect bioavailability and potential degradation, supplementing with fermentation-produced riboflavin through premixes is necessary to meet the vitamin requirements in pig nutrition [17, 38]. The data on riboflavin bioavailability from various feedstuffs in swine is limited, and further research is necessary in this area. Baker and Stein [39] referenced existing literature, noting that the bioavailability of riboflavin in a corn-soybean meal-based diet was approximately 60% of that found in crystalline riboflavin. They also highlighted that several dietary factors, such as excess levels of tetracycline, zinc, iron, copper, and ascorbate, may antagonize riboflavin, potentially impairing its absorption or utilization.

**Table 1:** Average vitamin B_2_ content in some feedstuffs for swine

Feedstuff	Vitamin B_2_, mg/kg	Reference
Corn	0.99	[40]
Oat	1.3–1.7	[41]
Wheat	0.8	[40]
Barley	1.5	[42]
Beer yeast (powder)	20.4	[40]
Brewers yeast	37.7	[43]
Soybean meal (oil < 5%, 48% protein )	2.9	[42]
Pea seeds	1.8	[41]
Rapeseed meal (oil < 5%)	3.8	[42]
Sunflower meal	3.0-3.6	[41]
Canola meal (oil < 5%)	3.8	[42]
Fish meal	3.4–8.1	[43]
Sugarbeet molasses	2.2	[44]
Sugarcane molasses	2.6	[44]

Upon ingestion, most of the coenzyme forms of riboflavin (FAD and FMN) are released from feed matrices in the stomach through the action of gastric acid and pepsin [45]. These noncovalently bound coenzymes are subsequently broken down into riboflavin by nonspecific pyrophosphatases and phosphatases in the proximal gut [45]. Industrially produced riboflavin, being in a non-esterified form, can be directly absorbed without the need for a hydrolysis step, unlike most of the plant-derived native vitamin B_2_ [13].

The transport of riboflavin across the intestinal epithelium involves several steps. Initially, vitamin B_2_ binds to the luminal surface of the enterocytes [46]. Riboflavin absorption occurs predominantly through a saturable, carrier-mediated process facilitated by the riboflavin transporter 3 (RFVT3) and, to a lesser extent, by passive diffusion when dietary concentrations are high [21, 47, 48]. Once inside the enterocytes, riboflavin may be phosphorylated to FMN by the enzyme flavokinase, a process requiring ATP [49]. FMN can be further converted to FAD by FAD synthetase [50]. However, the majority of free riboflavin, FMN, and FAD enter the portal circulation and are transported in plasma bound to albumin and immunoglobulins [51].

Upon entering the portal blood, riboflavin is transported to the liver, the central organ for nutrient metabolism [21]. Vitamin B_2_ in mammals is not stored in the body to a significant extent. Any excess intake that exceeds the renal reabsorption capacity is excreted in the urine as riboflavin itself or as its metabolites, such as 7-alpha-hydroxy riboflavin, 10-hydroxyethylflavin, and lumiflavin [52].

**Fig. 3:**
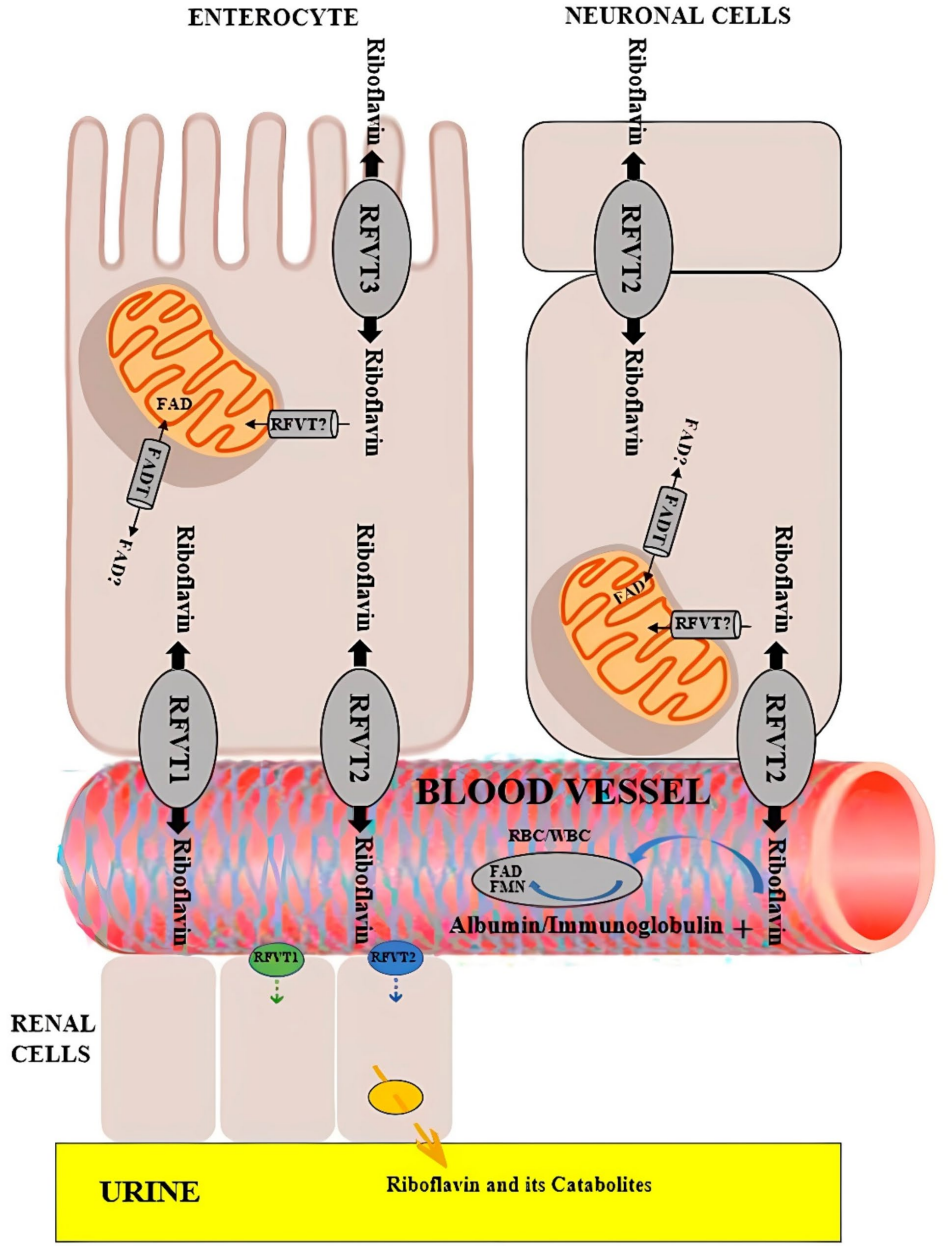
Metabolism and transport of riboflavin and flavocoenzyme [21]. Dietary flavin adenine dinucleotide (FAD) and flavin mononucleotide (FMN) are converted to riboflavin by non-specific hydrolases on the brush-border membrane of ileal enterocytes and are subsequently absorbed into the intestine via apically expressed RFVT3 (Riboflavin Transporter 3). Inside the enterocytes, riboflavin can either be further metabolized to FMN by riboflavin kinase and subsequently to FAD by FAD synthase or released into portal blood by basolaterally expressed RFVT1 and RFVT2 (Riboflavin Transporters 1 and 2). Circulating plasma riboflavin associates with albumin or globulins or is converted into a coenzyme form in erythrocytes or leukocytes. RFVT2-mediated transport allows riboflavin uptake into the brain where it is highly expressed, and additionally into endocrine organs, such as pancreas, liver, and muscle tissue. The mechanism of import of riboflavin into the mitochondrial matrix has not been precisely elucidated to date. “RFVT?” (Riboflavin Transporter) is depicted as a putative riboflavin transporter responsible for this step. The mitochondrial flavin adenine dinucleotide transporter (FADT) imports FAD from the cytosol into the mitochondria. The question mark indicates that FADT-mediated efflux of FAD from the mitochondrial matrix to the cytosol remains to be established

The liver, heart, and kidneys can store a limited amount of riboflavin, but it is generally distributed to other tissues as needed [48]. The systemic distribution of riboflavin to peripheral tissues occurs via the bloodstream [53]. In plasma, riboflavin binds to albumin and other plasma proteins, facilitating its transport to target tissues [54]. The cellular uptake of riboflavin involves its transport across the plasma membrane through specific riboflavin transporters [55]. This process starts with riboflavin’s release from the plasma via riboflavin binding protein (RBP) and involves a calcium-ion-dependent RBP receptor located in clathrin-coated pits on the phospholipid bilayer [56]. This receptor facilitates the endocytosis of riboflavin, allowing for its internalization and release inside the cell [55]. Afterward, both the receptor and RBP are recycled, while endosomes handle the catabolic processes. The enzymatic conversion of riboflavin into its active coenzyme forms, FMN and FAD, begins with the phosphorylation of riboflavin by riboflavin kinase, producing riboflavin 5’-phosphate [57]. This phosphorylation is essential for the subsequent synthesis of FAD from FMN (riboflavin 5’-phosphate) and ATP, a reaction catalyzed by FAD synthetase [18, 19]. Figure 3 provides an overview of the metabolism and transport of riboflavin, focusing on neuronal cells as an exemplary target. Table 2 outlines the primary biological functions of riboflavin in swine.

**Table 2:** Functions of riboflavin. (adapted from Shastak and Pelletier [13])

Function	Description
Energy production	Riboflavin is essential for producing energy via two key coenzymes, flavin mononucleotide (FMN) and flavin adenine dinucleotide (FAD).
Redox balance and antioxidant capacity	Riboflavin possesses indirect antioxidant properties, aiding in the neutralization of harmful free radicals within the body.
Metabolism of fats, nucleotides, amino acids, vitamins and steroid hormones	Riboflavin participates in the enzymatic reactions associated with the metabolism of lipids, amino acids, nucleotides, vitamins, and hormones.
Cellular function, growth, and development	Riboflavin plays a fundamental role in the regulation of cellular functions, growth, and developmental processes.
Reproductive functions	Riboflavin is essential for the reproductive performance. It affects fertility and embryonic development.
Nerve function	Riboflavin deficiency has been associated neurologic abnormalities related to peripheral neuropathy; in the central nervous system, vitamin B_2_ contributes to the regulation of neurohormones and biogenic amines.
Cell signaling	Riboflavin is essential for cell signaling by serving as a cofactor for nitric oxide synthase, facilitating the production of nitric oxide, which then acts on various tissues.

### Riboflavin requirements

Riboflavin absorption, utilization, and requirements in swine and other mammals are influenced by a variety of factors including age, gender, climatic conditions, genetic strain, health status, and diet composition [6, 53]. Young, growing pigs have elevated riboflavin requirements due to their rapid growth and high metabolic rates [58, 59]. As a result, their intestinal absorption capacity and tissue uptake mechanisms for riboflavin are particularly active. Health conditions, such as gastrointestinal diseases, can impair the absorption of riboflavin and other micronutrients by damaging the intestinal mucosa or altering the expression of riboflavin transporters [60, 61]. Additionally, antinutritional factors in the diet, such as certain fibers, can potentially bind riboflavin and its coenzymes, thereby reducing its bioavailability [62]. Reproductive sows, particularly those that are lactating, also have increased riboflavin requirements to support milk production and maintain their own health [5, 63, 64].

To ensure that swine meet their riboflavin needs, diets are typically formulated to include adequate levels of this vitamin. While natural feed ingredients such as grains and legumes contain some vitamin B_2_, they often do not provide sufficient amounts to meet the needs of growing or breeding swine [17]. Therefore, riboflavin supplements are commonly added to feed to ensure pigs receive adequate quantities [38].

Research continues to refine our understanding of vitamin requirements in swine, with recent studies having explored optimal levels for different production stages [65–67]. Scientific committees such as the National Academies of Sciences, Engineering, and Medicine (NASEM) [68] provide nutrient and micronutrient requirement estimates that serve as general guidelines for practical use (Table 3). However, it is crucial to recognize that these guidelines are based on ideal conditions and may not reflect individual variations in vitamin requirements, which can be influenced by genetics, age, sex, body weight, and other contextual factors. Consequently, estimates derived from controlled research settings may have limitations when applied to real-world scenarios, such as varying stocking densities, disease pressures, and genetic differences [8].

**Table 3:** NASEM vitamin B_2_ requirement estimates

Committee	Vitamin B_2_ requirement estimates, mg/kg feed			
	Piglets	Finishing gilts and barrows	Gestating gilts and sows	Lactating gilts and sows
NASEM 1944^*^	-	1.7	-	-
NASEM 1950^*^	-	1.8–1.9	1.85	1.85
NASEM 1953^*^	2.7	2.2	2.7	2.7
NASEM 1959^*^	3.1–3.3	2.2–2.7	3.3	3.3
NASEM 1964^*^	3.1–3.3	2.2–2.7	3.3	3.3
NASEM 1968^*^	3.0	2.2–2.6	3.3–4.1	3.3–4.1
NASEM 1973^*^	3.0	2.2–2.6	3.5-4.0	3.5-4.0
NASEM 1979^*^	3.0	2.2–2.6	3.0	3.0
NASEM 1988 [69]	3.0–4.0	2.0-2.5	3.75	3.75
NASEM 1998 [41]	3.0–4.0	2.0-2.5	3.75	3.75
NASEM 2012 [68]	3.0–4.0	2.0-2.5	3.75	3.75

^*^Adapted from Kim and Lindemann [70]

While riboflavin requirements may increase due to stress, infection, illness, poor hygiene, or temperature-related stress, these factors are not typically considered in nutritional physiological requirements. However, they must be addressed in practical settings by ensuring adequate riboflavin levels in feed and regularly monitoring pig health and performance [71]. This enables nutritionists and feed formulators to make necessary dietary adjustments to meet the pigs’ nutritional needs. It is evident from Table 4 that average industrial vitamin B_2_ fortification levels are higher than those set by NASEM [68].

Recent studies have shown that providing vitamin levels beyond the NASEM [68] requirements can enhance swine performance and carcass quality [65–67, 71, 76]. Since the NASEM requirement estimates for riboflavin have remained unchanged for many decades, this suggests that the data used to derive these estimates - particularly those evaluating the impact of vitamin B_2_ on various physiological parameters - may be outdated. Therefore, conducting comprehensive research to reassess the actual riboflavin requirements in swine across all stages of production is both scientifically justified and timely. To maximize animal productivity, it is essential to optimize feed intake and ensure that dietary requirements are met at all production stages. Recommendations for vitamin B_2_ levels in feed from pig breeding companies can guide practical feeding strategies. These guidelines are based on scientific research and data and are likely to include certain safety margins. The Table 5 provides riboflavin recommendations from various pig breeding companies.

With current prices for vitamin B_2_ (80% grade) at 17 euros per kilogram and an inclusion level of 6 g of pure riboflavin per metric ton of feed - two times higher than NASEM estimates - the contribution of vitamin B_2_ per ton amounts to 0.13 euros. In comparison to an average swine feed cost of 400 euros per ton, this represents just 0.03% of total feed costs, which can be considered negligible. However, despite this minimal commercial contribution, riboflavin supplementation plays a significant role in enhancing swine productivity, highlighting the necessity of adequate dietary levels.

Riboflavin exhibits a low toxicity profile, and toxicological studies indicate that it does not pose safety concerns for target animals when used within current recommended levels [82, 83]. In one study, groups of 50 male and female Sprague–Dawley rats were administered single doses of up to 15,000 mg/kg body weight of riboflavin (80%) via oral gavage. No increase in mortality, nor any notable changes in behavior or clinical appearance, were observed over the following seven days, leading to the conclusion that the acute oral toxicity of vitamin B_2_ is low [82].

**Table 4:** Average industrial supplementation levels of vitamin B_2_ in the USA, China, Brazil, and Denmark. (sources: Dalto and Da Silva [72]; Yang et al. [73]; Kjeldsen [74]; Faccin et al. [75])

Diet	USA (mg/kg feed)			Brazil (mg/kg feed)		Denmark (mg/kg feed)		China (mg/kg feed)		
	Average		Ratio to NASEM*	Average	Ratio to NASEM*	Average	Ratio to NASEM*	Average	Ratio to NASEM*	
Creep diets (weaning to 8 kg)								6.0		1.5
Creep diets (3 to 20 days of age)				8.3	2.1					
Weaning diet (from 6.5 kg)						10.0	2.9			
Nursery diet 1 (weaning to 7 kg)	10.6	2.7								
Nursery diets I (21 to 35 days of age)				6.5	1.7					
Early nursery diets (8 to 15 kg)								5.8		1.7
Nursery diets II (36 to 49 days of age)				6.2	1.8					
Nursery diet 2 (7 to 11 kg)	8.2	2.3								
Weaners (9–20 kg)						4.5, 9.6^1^	1.5, 3.2^1^			
Late nursery diets (15 to 25 kg)								5.0		1.7
Nursery diets III (50 to 70 days of age)				5.3	1.8					
Nursery diet 3 (11 to 23 kg)	6.6	2.2								
Growing diets (71 to 120 days of age)				3.9	1.3					
Growing diets (25 to 60 kg)								3.4		1.4
Finishing diets (121 days of age to slaughter)				3.3	1.7					
Early-finishing diets (23 to 55 kg)	4.9	2.0								
Early-finishing diets (60 to 90 kg)								3.2		1.6
Mid-finishing diets	4.2	2.1								
Finishers (30–100 kg)						2.2, 2.1^2^	1.1, 1.1^2^			
Late finishing diets (90 kg to market)								3.2		1.6
Late finishing diets (100 kg to market)	3.6	1.8								
Gilt-development diets	7.7	2.1		6.5	1.8			4.6		1.3
Breading stock						5.3	1.4			
Gestation diets	8.6	2.3		5.5	1.5	5.3	1.4	4.7		1.3
Transition diets				3.9	1.0					
Lactation diets	8.6	2.3		5.5	1.5			4.7		1.3
Boar diets	8.9	2.4		6.1	1.5			4.7		1.3

*Values represent average supplementation rates as a proportion to total dietary vitamin requirement estimates from the NASEM [68]; ^1^Low protein diet; ^2^with 200% phytase.

**Table 5:** Vitamin B_2_ recommendations for some pig breeds

Breed or organisation	Vitamin B_2_ recommendation, mg/kg feed				
	Piglets	Finishing gilts and barrows	Gestating gilts and sows	Lactating gilts and sows	Source
PIC	13.0	4.9–5.7	10.0	10.0	[77]
Genesus	12.0	4.7-5.0	10.0	10.0	[78]
Topigs	7.5–15.0	5.0–8.0	4.0–5.0	5.0-7.5	[79]
Hypor	Min. 7.5	Min. 3.5-4.0	5.0	7.5	[80, 81]

Additionally, another EFSA opinion reported that vitamin B_2_ produced by Ashbya gossypii was found to be free from mutagenic or genotoxic potential. The EFSA FEEDAP Panel concluded that riboflavin and riboflavin 5’-phosphate sodium are safe for target animals, including swine, with a wide margin of safety—estimated to be 20 to 60 times higher than practical usage levels [82, 83].

### Riboflavin in swine well-being across various physiological stages

#### Suckling piglets

During the suckling phase, riboflavin plays a critical role in supporting growth and metabolic processes. Adequate riboflavin levels are essential for the development of the nervous system and for various cellular functions [84, 85]. However, research on the effects of vitamin B_2_ in suckling piglets remains limited. Studies in suckling rats have shown that riboflavin deficiency leads to a reduced growth rate and an increased activation coefficient of erythrocyte glutathione reductase [86]. Additionally, riboflavin deficiency was associated with a decrease in whole-body oxygen consumption per unit of body weight, particularly following noradrenaline stimulation. To ensure adequate riboflavin transfer to piglets via colostrum and milk, it is crucial to fortify maternal diets with this essential micronutrient. However, the precise vitamin B_2_ requirements for suckling piglets have not yet been fully determined and require further research to be accurately established.

#### Weaned piglets

As piglets transition to solid feed, riboflavin continues to be vital for immune function and overall health [7, 87]. The weaning phase and the period following it are marked by rapid growth potential, disturbed feed intake, changes in metabolism, and increased vulnerability to health issues, making proper nutrition, including vitamin supplementation, essential for piglet well-being and development. Nursery diets typically include sources of riboflavin to promote optimal growth rates and improve feed efficiency. Deficiencies during this stage can lead to poor growth and increased susceptibility to diseases [7, 88, 89]. Based on recommendations from four major breeding companies, the dietary riboflavin levels for piglets are advised to range between 7.5 and 15 mg per kilogram of feed, depending on the breed.

#### Growing pigs

In growing pigs, riboflavin supports energy metabolism and enhances the efficiency of nutrient utilization [4, 58, 90]. It is particularly important during rapid growth phases, influencing muscle development and overall weight gain. Ensuring adequate riboflavin levels in the diet can improve performance and production efficiency [58, 59]. According to guidelines from four leading breeding companies, the dietary riboflavin levels for growing pigs are advised to range between 4.0 and 12.0 mg per kilogram of feed, depending on the breed.

#### Finishing pigs

For finishing pigs, riboflavin contributes to energy metabolism and supports the development of lean muscle mass. A balanced intake of vitamin B_2_ and other B vitamins during this stage is required for achieving optimal feed conversion ratio, carcass yield, and overall performance [65]. Based on recommendations from four major breeding companies, the dietary vitamin B_2_ levels for finishing pigs are advised to range between 3.5 and 8.0 mg per kilogram of feed, depending on the breed.

#### Gestating gilts and sows

In gestating sows, riboflavin is critical for fetal development and placental health [91]. Adequate vitamin B_2_ intake supports proper embryonic growth and minimizes the risk of reproductive disorders [92]. Supplemental riboflavin in gestating gilt and sow diets helps ensure the health of both the sow and her offspring [64]. According to guidelines from four leading breeding companies, the dietary riboflavin levels for gestating gilts and sows are advised to range between 4.0 and 10.0 mg per kilogram of feed, depending on the breed.

#### Lactating gilts and sows

During lactation, riboflavin plays a vital role in milk production and quality [93–95]. It supports the nutritional needs of piglets during their early growth stages. Adequate riboflavin levels in the lactating sow’s diet are essential for maximizing milk yield and promoting healthy piglet development. As per the recommendations from four leading breeding companies, the suggested dietary levels of vitamin B_2_ for lactating gilts and sows should fall within a range of 5.0 to 10.0 mg per kilogram of feed, with variations based on breed.

#### Boars

Vitamin B_2_ is essential for the reproductive health of boars, influencing sperm function, antioxidative status, and spermatogenesis [96]. Ensuring that boars receive adequate riboflavin is important for optimizing breeding performance and overall reproductive success. Based on recommendations from four major breeding companies, the dietary vitamin B_2_ levels for sexually active boars are advised to be approximately 10.0 mg per kilogram of feed.

In summary, riboflavin is a vital micronutrient for swine across all life stages. Adequate riboflavin intake not only supports individual growth and development but also enhances overall herd health and productivity. Nutritional strategies should be implemented to ensure that riboflavin levels meet the requirements specific to each physiological stage, promoting optimal performance and health in swine production.

### Consequences of riboflavin deficiency (Ariboflavinosis)

Riboflavin is a standard component in premixes for commercially produced swine feed, which significantly reduces the incidence of hypovitaminosis B_2_ in conventional swine farming. However, weaned piglets remain at risk due to their typically reduced feed intake following weaning. Challenges also arise in organic swine production, where the use of microbially produced vitamin B_2_ is prohibited. Furthermore, in conventional farming, deficiencies of riboflavin typically occur due to errors in premix or feed formulation, such as the inadvertent omission of vitamin B_2_ during the mixing process.

One of the most notable consequences of ariboflavinosis in swine is impaired growth. Vitamin B_2_ as part of many enzymes is crucial for the metabolism of carbohydrates, fats, and proteins [5]. Inadequate riboflavin disrupts these metabolic processes, leading to poor feed conversion and reduced growth rates [14, 90]. Young pigs, in particular, exhibit stunted growth when deprived of sufficient riboflavin, as their rapidly developing bodies require efficient energy production and nutrient utilization [58, 88, 89]. The deficiency can manifest as reduced appetite, slower weight gain, and weight loss [6]. This not only affects the individual animal’s health but also results in economic losses for farmers.

Reproductive performance in swine is also significantly affected by riboflavin deficiency. Vitamin B_2_ is vital for the proper functioning of reproductive tissues and the maintenance of pregnancy. Sows deficient in riboflavin often experience premature farrowing, increased occurrences of stillborn piglets, neonatal death, and insufficient milk production during lactation [5, 63, 97]. The health of piglets born to riboflavin-deficient sows is often compromised, exhibiting signs of weakness and poor vitality, which can impact overall litter performance [5].

Vitamin B_2_ deficiency also has profound effects on the nervous system of swine. Riboflavin is involved in the synthesis of coenzymes necessary for the proper functioning of the nervous system [98]. Deficient animals may display neurological symptoms such as impaired neurological development, disturbances in neurological processes, and degeneration of the myelin sheath of the sciatic and brachial nerves [41, 68, 89, 99, 100]. These symptoms are particularly evident in young pigs and can severely compromise their ability to thrive.

The skin and mucous membranes of swine are also adversely affected by hypovitaminosis B_2_. Riboflavin plays a role in maintaining the integrity of skin and mucosal surfaces, and its deficiency can lead to lesions and dermatitis [89]. Affected pigs may exhibit mottled erythematous eruptions of the skin, particularly around the snout, behind the ears, along the midline of the back, in the groin area, and over the abdomen [101]. The affected skin becomes scaly, and reddish-brown scabs are formed. These eruptions not only cause discomfort and pain to the animals but also make them more susceptible to secondary infections.

The immune system of swine is another area that suffers due to riboflavin deficiency. Vitamin B_2_ is essential for maintaining an effective immune response, and its insufficiency can lead to immunosuppression [7]. Research on a mouse monocyte/macrophage cell line indicates that riboflavin deprivation can impair macrophage activity and viability, reducing their ability to respond to bacterial or yeast-derived factors like lipopolysaccharide and zymosan [102]. Short-term deprivation (5 days) led to abnormal macrophage activation, lower cell viability, and increased release of tumor necrosis factor-α (TNF-α) and high-mobility group box 1 (HMGB1). It also decreased levels of inducible nitric oxide synthase (iNOS), nitric oxide (NO), and key immune proteins. However, supplementing riboflavin (300 nM) on the third or fourth day effectively reversed these changes.

Pigs with inadequate riboflavin intake often exhibit a weakened immune system, making them more susceptible to infections and diseases [68]. This increased susceptibility can lead to higher morbidity and mortality rates, as the animals are less capable of mounting an effective defense against pathogens. In commercial swine production, this can result in significant economic losses due to increased veterinary costs, higher mortality rates, and reduced overall herd health.

Another significant consequence of riboflavin deficiency in swine is its impact on ocular health. Riboflavin is indirectly involved in maintaining the health of the eyes, and a deficiency can lead to conditions such as lens opacities, cataracts and other vision problems [101, 103]. Swine with ariboflavinosis may show signs of photophobia (sensitivity to light) [89]. These ocular conditions not only cause discomfort and pain to the animals but can also impair their ability to locate feed and water, further exacerbating nutritional deficiencies and impacting their overall health and well-being.

Moreover, riboflavin deficiency can affect the liver function of swine. The liver is a key organ in detoxification processes and the metabolism of nutrients. Vitamin B_2_ is involved in the functioning of various liver enzymes that are crucial for these processes [104]. Ariboflavinosis causes liver lipid accumulation, probably by impairing fatty acid β-oxidation and electron transport chain function [41, 58, 105]. Hypovitaminosis B_2_ can impair liver function, leading to an accumulation of toxins in the body and further exacerbating health problems in swine [106].

### Effects of riboflavin supplementation

#### Piglets

Qin et al. [4] investigated the impact of riboflavin on the intestinal development and function of weaned piglets. They divided 21 piglets, weaned at 21 days of age, into three groups receiving 0 mg/kg, 3.5 mg/kg, or 17.5 mg/kg riboflavin in their diets for 28 days. The group receiving 3.5 mg/kg riboflavin showed the best feed conversion ratio. Villus height and the ratio of villus height to crypt depth in duodenum were lower in the riboflavin-free group compared to the other two groups (*P* < 0.05). However, no significant differences were observed in the heights of jejunal and ileal villi, nor in the depths of crypts in the duodenum, jejunum, and ileum across the three treatment groups (*P* > 0.05). Additionally, the ratio of villus height to crypt depth in the jejunum and ileum did not vary significantly among the treatments. Transcriptomic analysis in duodenal mucosal molecules revealed significant enrichment in apoptosis pathways for the riboflavin-free group and in pro-inflammatory pathways for the high riboflavin group. The riboflavin-free group also had the lowest (*P* < 0.05) total antioxidant capacity and glutathione peroxidase activity. Overall, riboflavin levels appear to influence intestinal morphology and function, as evidenced by changes in villus height and crypt ratios in the duodenum, along with alterations in antioxidant capacity and apoptosis pathways in the riboflavin-free group. These results suggest that vitamin B_2_ plays a role in maintaining intestinal integrity and immune balance, with potential implications for nutrient absorption and gut health.

The selenium-dependent glutathione peroxidase (GSH-Px) system is essential for protecting pig tissues from peroxidation [107]. It requires several key nutrients: selenium as a component of GSH-Px; cysteine, a sulfur-containing amino acid necessary for GSH synthesis; riboflavin for FAD, which supports glutathione reductase activity; and niacin, which is a precursor for NADPH [108]. Together, these nutrients maintain the antioxidant defense system in pigs. Parsons et al. [108] studied the effects of dietary riboflavin and selenium source on the performance and selenium metabolism of weanling pigs. Animals fed a diet supplemented with 10 mg/kg of riboflavin showed faster growth compared to those on a riboflavin-unsupplemented diet. The percentage of active erythrocyte glutathione reductase declined significantly (*P* < 0.01) when pigs were on the riboflavin-unsupplemented diet and remained lower than in riboflavin-supplemented pigs after 12 days. Vitamin B_2_ supplementation increased glutathione peroxidase activity in the kidneys and muscles, and influenced selenium distribution, resulting in higher selenium concentrations in the liver and heart and lower plasma levels. However, the activity of glutathione peroxidase in the heart and brain was not influenced by the dietary levels of vitamin B_2_. Additionally, riboflavin supplementation and the type of selenium source did not affect the apparent absorption of selenium. Nevertheless, riboflavin supplementation did reduce urinary selenium excretion, with selenium retention differing depending on the specific form of selenium consumed. Finally, the concentrations of muscle and brain tissues were not significantly affected by vitamin B_2_ supplementation.

In a study by Brady et al. [107], one-week-old piglets were assigned to three dietary treatments for six weeks: a riboflavin-free diet, a riboflavin-supplemented diet with 25 mg/kg, and a pair-fed group where half of the supplemented pigs were matched to the deficient pigs’ feed intake. At the study’s conclusion, blood and organ samples were collected from the animals. The study found a 75% reduction in erythrocyte glutathione reductase activity in pigs on the riboflavin-free diet, with no change in reduced glutathione levels. Ariboflavinosis led to significant decreases in hepatic glutathione peroxidase activity and selenium concentration, while muscle glutathione peroxidase activity also decreased, though muscle selenium levels remained unaffected.

Lutz and Stahly [6] conducted a study with six experimental treatments, involving two genetic strains of pigs (high versus moderate lean) and three dietary riboflavin levels (0, 3.7, 7.4 mg/kg). Pigs, weaned at 8 to 12 days of age, were housed in a nursery unit and fed a low riboflavin basal diet until they reached 10 kg body weight. They were then assigned to one of the riboflavin treatments, and their weight and feed consumption were measured at four-day intervals until they reached 26 kg. Blood samples were analyzed to assess erythrocyte glutathione reductase activity. Riboflavin supplementation significantly improved (*P* < 0.05) gain/feed ratio for both strains. Erythrocyte glutathione reductase activity was numerically higher for the highest riboflavin supplementation level (7.4 mg/kg) in the high lean strain. Furthermore, the study revealed that the vitamin B_2_ needs for body protein accretion were six times higher than those for body fat accretion, with daily requirements ranging from 5.2 to 10.8 mg. These findings suggest that riboflavin requirements for pigs are significantly greater than NASEM [41] estimates for 10 to 20 kg pigs.

**Fig. 4:**
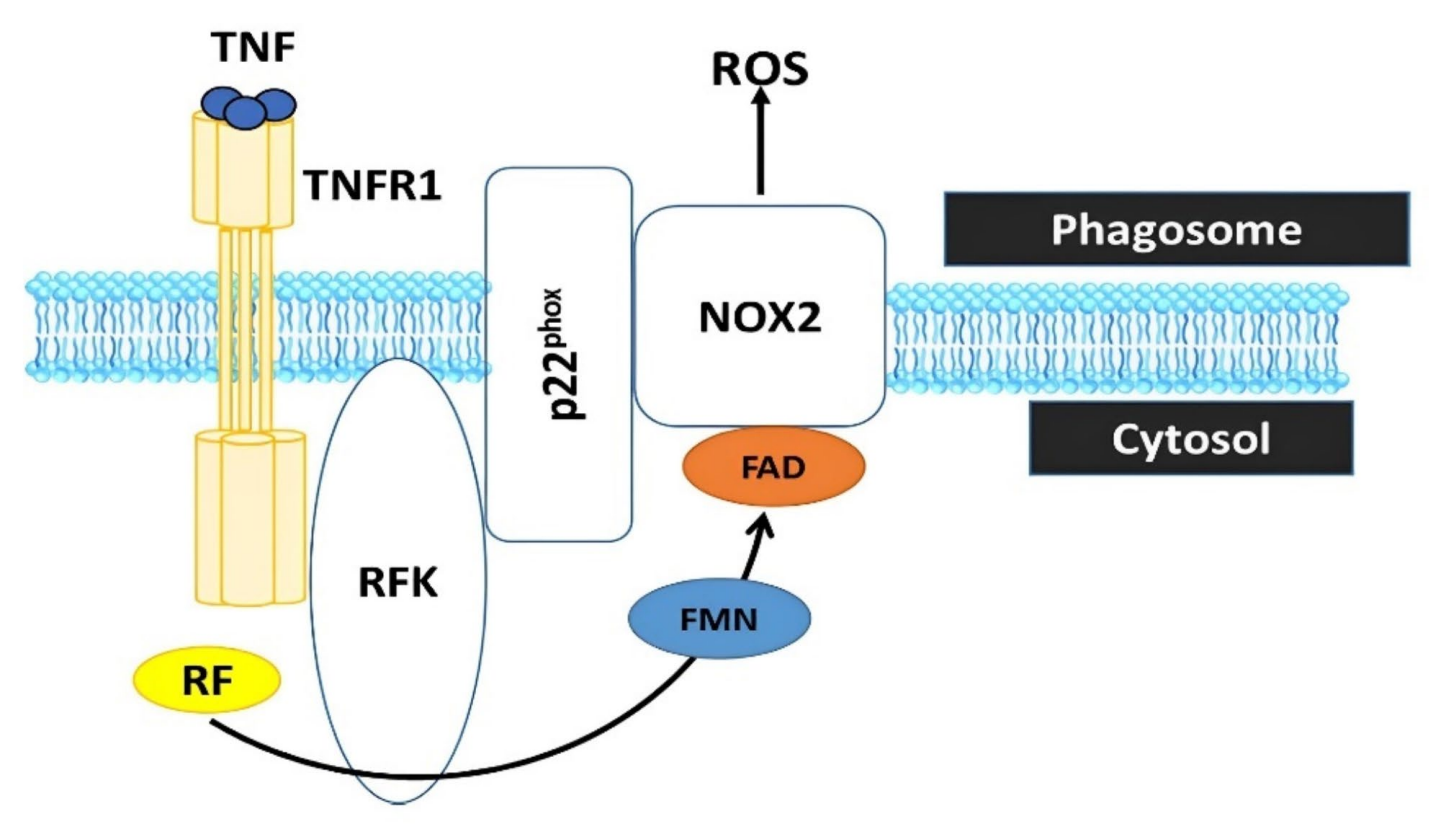
Riboflavin is converted by riboflavin kinase into flavin monophosphate (FMN) and flavin adenine dinucleotide (FAD), which are essential cofactors of the phagocytic NADPH oxidase 2 (Nox2) to generate reactive oxygen species (ROS). Riboflavin deficiency renders the phagocyte Nox2 incapable of producing ROS, a process crucial for deactivating phagocytosed microbes and regulating the inflammatory response in innate immune cells [7]. TNF = tumor necrosis factor; TNFR1 = tumor necrosis factor receptor 1

In an earlier study, Mitchel et al. [58] investigated riboflavin requirements at temperatures of 29 °C and 6 °C. Pigs were deprived of riboflavin for three weeks on a semi-synthetic diet with low riboflavin content, then fed eight varying levels of riboflavin (from 0 to 5 mg/kg) for 7 or 8 weeks. Feed consumption was regulated, except when pigs on lower riboflavin levels showed deficiency symptoms. The results clearly indicated that the vitamin B_2_ requirement increased by a factor of 2.9 at the lower temperature. The study found that riboflavin deficiency was associated with a significant increase in the concentration and percentage of neutrophilic granulocytes in the blood. This change in blood cell composition suggests potential impacts on immune function, as elevated neutrophil levels may indicate a response to underlying immune system stress or inflammation. Suwannasom et al. [7] recently elucidated how riboflavin positively influences immune function in mammals (Fig. 4).

#### Breeding swine

The importance of riboflavin in reproduction is demonstrated by a recent case study reported by Torreggiani et al. [5], which involved two organic farms with 320 sows (Farm A) and 250 sows (Farm B). These farms experienced high rates of premature farrowing, weak-born or stillborn piglets, and severe intra-litter mortality (60–100%) between 2019 and 2020. Initial investigations into infectious causes, water quality, and general feed composition showed no significant results. A more detailed analysis revealed critically low riboflavin levels in the feed, at 1.25 mg/kg, compared to the NASEM minimum recommended level of 3.75 mg/kg. Supplementing with riboflavin one month before farrowing (30 mg i.m.) resolved the issues rapidly, eliminating stillbirths and intra-litter mortality, thereby confirming riboflavin deficiency as the underlying cause and highlighting its critical role in reproductive health.

Esch et al. [109] conducted a trial with 20 crossbred cycling gilts, assigning them to either a riboflavin-adequate (4.1 mg/kg mg/kg) or a riboflavin-deficient (0.8 mg/kg) diet. The gilts were monitored daily for estrus, and blood samples were collected bi-weekly to measure progesterone and estradiol-17β levels. After 28 days, urine samples were analyzed for estrone sulfate. The gilts on the riboflavin-deficient diet became anestrous approximately 63 days into the trial, with an increased interval between estrous periods. Compared to the controls, the riboflavin-deficient gilts exhibited significantly higher progesterone (64.2 ± 1.0 ng/ml, *P* < 0.001) and estradiol-17β levels (82.6 ± 1.8 pg/ml, *P* < 0.001), while estrone sulfate levels were substantially lower (4.1 ± 0.8 ng E1 S/mg creatinine, *P* < 0.001), indicating disrupted ovarian cyclicity and abnormal sex steroid profiles.

Frank et al. [97] conducted a study with 25 crossbred gilts and 25 crossbred sows to determine the riboflavin requirements of lactating swine. The females were fed a 12% crude protein corn-soybean meal diet, which was marginally deficient in riboflavin (2.3 mg/kg) during gestation. After farrowing, the sows and gilts were given one of five riboflavin-supplemented lactation diets (ranging from 1.25 to 5.25 mg/kg) for 24 days, with piglets not receiving supplemental feed. Erythrocyte glutathione reductase activity coefficients (EGRAC) measured on days 1 and 24 postpartum revealed that gilts and their piglets had higher EGRAC values compared to sows and their piglets, indicating significant treatment and age interactions. Notably, gilts on the diet with 1.25 mg/kg riboflavin experienced higher piglet mortality, consumed less feed, and lost more weight compared to sows on the same diet, despite both groups having similar riboflavin requirements estimated at approximately 16.2 to 16.3 mg/day.

Pettigrew et al. [92] evaluated the impact of riboflavin levels on 118 sows at two farms. The sows were fed diets providing 10 (control), 60, 110, or 160 mg of riboflavin per day (5.6, 33.3, 61.1 or 88.9 mg of riboflavin per kg of diet, respectively) for 21 days starting at breeding, with the control level used for the remainder of the reproductive cycle. The farrowing percentage tended to be higher for sows fed higher vitamin B_2_ levels compared to the control group (66.7% for 10 mg/day versus 85.7%, 93.3%, and 86.7% for 60, 110, and 160 mg/day, respectively; *P* < 0.10). The authors concluded that high riboflavin supplementation in the sows’ diet during early pregnancy may improve the farrowing rate but does not affect litter size.

To evaluate the effects of increased dietary riboflavin on pregnant swine, Bazer [64] conducted an experiment with 14 control and 15 riboflavin-fed gilts. The gilts received either 0 or 100 mg of riboflavin per head and day (0 or 45.5 mg of supplemental riboflavin per kg of diet, respectively) from days 4 to 10 of gestation and were hysterectomized on day 30. Results showed that riboflavin-fed gilts had higher live embryo counts (13.2 vs. 11.5; *P* = 0.07), better embryonic survival (84% vs. 75%; *P* < 0.05), and increased allantoic fluid volumes (235 ml vs. 200 ml; *P* < 0.05). In another study with 48 control (0 mg per head and day or 0 mg of supplemental riboflavin per kg of diet) and 51 riboflavin-fed sows (100 mg per head and day or 45.5 mg of supplemental riboflavin per kg of diet), those receiving riboflavin had a higher farrowing rate (80.4% vs. 70.8%; *P* < 0.05) and improved reproductive outcomes. Vitamin B_2_-fed sows had greater numbers of piglets born, more piglets alive at days 21 and 42, and higher total litter weights at these times. The summary in Table 6 presents the effects of riboflavin supplementation on swine.

**Table 6:** Impact of riboflavin supplementation on the performance, antioxidant capacity, and immunity of swine

Animal	Supplemental riboflavin levels	Effects of riboflavin supplementation	Reference
Weaned piglets	0, 3.5, or 17.5 mg/kg feed	Improved feed conversion ratio, improved villus height and the ratio of villus height to crypt depth, higher total antioxidant capacity and glutathione peroxidase activity.	[4]
Weaned piglets	0 or 10 mg/kg feed	Improved body weight, higher percentage of active erythrocyte glutathione reductase, increased glutathione peroxidase activity in the kidneys and muscles, and higher selenium concentrations in the liver and heart.	[108]
Weaned piglets	0 or 25 mg/kg feed	Increase in erythrocyte glutathione reductase activity, increase in hepatic glutathione peroxidase activity and selenium concentration.	[107]
Weaned piglets	0, 3.7 or 7.4 mg/kg feed	Improved body weight gain and gain/feed ratio, numerically higher erythrocyte glutathione peroxidase activity.	[6]
Weaned piglets	From 0 to 5 mg/kg feed	Significantly lower concentration and percentage of neutrophilic granulocytes in the blood	[58]
Weaned piglets	0 or 1.5 mg per head and day	Higher growth rate and feed intake.	[89]
Growing swine	1.2 or 1.8 mg/kg feed	Improved growth rate and feed efficiency.	[110]
Gestating sows	1.25 mg/kg feed or 30 mg i.m.	Elimination of stillbirths and intra-litter mortality.	[5]
Cycling gilts	0.77 or 4.1 mg/kg feed	Lower progesterone and estradiol-17β levels, while estrone sulfate levels were substantially higher, indicating normal ovarian cyclicity and sex steroid profiles.	[109]
Lactating gilts	1.25, 2.25, 3.25, 4.25 or 5.25 mg/kg feed	Lower piglet mortality, higher feed intake, and lower weight loss.	[97]
Gestating sows	10 (control), 60, 110, or 160 mg per head and day	Higher farrowing rate.	[92]
Gestating gilts	0 or 100 mg per head and day	Higher live embryo counts, better embryonic survival and increased allantoic fluid volumes.	[64]
Gestating sows	0 or 100 mg per head and day	Greater numbers of piglets born, more piglets alive at days 21 and 42, and higher total litter weights at days 21 and 42.	[64]
Gestating and lactating sows	1.2, 1.8, 2.8 or 3.6 mg/kg feed	Higher conception rate and increased litter size.	[63]
Boars	0, 5, 10, 15, 20 or 25 µM in sperm solution	Increased frozen-thawed sperm progressive motility, higher activities of superoxide dismutase, glutathione peroxidase and catalase, as well as decreased the malondialdehyde content .	[96]

### Research and future directions

Despite considerable research on riboflavin in swine nutrition over the past eight decades, several fundamental gaps in our understanding remain. Most notably, the precise vitamin B_2_ requirements for modern pig breeds across all stages of production have not been conclusively established, and data from older studies may no longer be fully reliable due to advancements in genetic selection, feed formulations, and production systems. Additionally, the bioavailability of riboflavin from various feedstuffs is not yet fully understood, which complicates decisions on nutritional intervention strategies.

Furthermore, there is limited information on riboflavin transfer from sows to piglets during gestation and lactation, through both the placenta and colostrum. These uncertainties raise important questions about the adequacy of current supplementation practices. Without a deeper understanding of these key aspects, it becomes challenging to determine the most effective riboflavin supplementation strategies.

While recent studies offer encouraging insights, much of the available knowledge is derived from studies that may be outdated. Consequently, more rigorous research is needed to provide a comprehensive and up-to-date understanding of riboflavin nutrition in swine. This includes studies to determine precise requirements, explore the bioavailability of riboflavin from diverse feed ingredients, and investigate its transfer from sows to piglets.

Another critical gap is understanding the interactions between riboflavin and other dietary components, such as vitamins, minerals, and anti-nutritional factors [111]. Gaining a deeper insight into these interactions is essential for optimizing swine diets to improve riboflavin absorption and utilization, thereby enhancing overall health and productivity.

Comprehensive studies on the long-term effects of subclinical ariboflavinosis are also needed. For instance, to investigate the long-term effects of subclinical hypovitaminosis B_2_ in swine, a well-designed longitudinal study could be implemented. The study would include a control group of pigs receiving slightly suboptimal levels of riboflavin, and a treatment group receiving adequate supplementation. Researchers would monitor key metrics such as growth rates, feed efficiency, and overall health from weaning through to slaughter, while routinely measuring blood riboflavin concentrations to assess adequacy levels. By analyzing these data, potential associations between riboflavin deficiency and impaired productivity could be identified, providing valuable insights to inform more precise and effective riboflavin supplementation strategies in swine production systems.

Additionally, detailed research is necessary to establish safety margins and identify early biomarkers of deficiency. Moreover, there is a lack of research on how environmental conditions, such as heat stress, and genetic factors influence riboflavin requirements and metabolism in swine [112]. These factors could significantly impact nutritional strategies for different farming systems and pig breeds.

A significant challenge remains in advancing precision nutrition with vitamins, primarily due to the limited understanding of exact vitamin requirements in porcine species. While future trends may explore advances in precision nutrition facilitated by big data and machine learning, it is essential to acknowledge the existing limitations in our knowledge [113–115]. Tailoring herd-specific nutritional recommendations requires a more robust database that integrates data on genetics, health status, and environmental conditions [116, 117]. Currently, this lack of comprehensive information hampers the ability of nutritionists to develop customized vitamin enrichment tactics that effectively maximize growth and productivity while minimizing waste and environmental impact. Recognizing these gaps and the complexity of determining accurate vitamin requirements is crucial for guiding future research and development in the field. Thus, while the potential for precision nutrition exists, we must clearly articulate the challenges that need to be overcome to realize this vision.

Developing and testing functional feed additives that enhance riboflavin absorption and utilization is another potential innovation. Additives such as probiotics, prebiotics, and enzyme blends could improve gut health and micronutrient uptake, ensuring that swine receive the maximum benefit from riboflavin and other vitamins in their diets [118].

Future research should focus on the environmental impact of riboflavin supplementation, as there may be misconceptions about its significance in this context. It is essential to consider both the carbon footprint associated with the production of riboflavin and the potential environmental effects of its use in animal production systems. Conducting life cycle assessments can provide valuable insights into the sustainability of various commercial riboflavin products and supplementation strategies. This approach will help identify practices that not only minimize the carbon footprint but also reduce the overall environmental burden associated with animal production, ultimately contributing to global sustainability efforts [119].

Investigating the broader health and welfare implications of vitamin B_2_ supplementation is crucial. Beyond growth performance, riboflavin’s role in immune function, disease resistance, and overall well-being needs to be explored [120, 121]. This holistic approach can lead to more resilient and healthier pig populations. As new technologies and ingredients are developed, ensuring regulatory compliance and consumer acceptance will be vital. Transparent communication of the benefits and safety of innovative riboflavin supplementation methods can facilitate their adoption in the industry [120].

Finally, microbial production of vitamin B_2_ presents a cost-effective and sustainable option, as it often utilizes renewable resources and can reduce energy demands. The controlled nature of microbial fermentation allows for consistent production yields, reducing variability and ensuring a stable supply of riboflavin for premix and feed manufacturers. Further enhancing microbial production of riboflavin through genetic engineering and fermentation technology offers a cost-effective alternative to chemical synthesis [122]. Optimized strains of bacteria and yeast can produce high levels of riboflavin, which can be incorporated into feed via premixes or used to enrich existing feed ingredients [123].

While current research has established the fundamental importance of riboflavin in swine nutrition, significant opportunities remain to refine and enhance our understanding and application of this essential vitamin. Addressing the identified knowledge gaps and embracing future trends and innovations will be key to optimizing vitamin B_2_ supplementation, improving swine health and productivity, and advancing the sustainability of the pork industry.

## Conclusion

Riboflavin is crucial for swine metabolism, development, and health. Deficiency can lead to impaired growth, reproductive issues, decreased feed efficiency, and compromised immune function, causing economic losses for farmers.

Swine riboflavin requirements vary with age, gender, climate, genetic strain, health status, and diet. Young pigs and lactating sows need more due to their rapid growth and high metabolism. To meet these needs, diets are universally supplemented with riboflavin.

In conclusion, optimizing riboflavin supplementation is essential for the sustainable development of the pig industry. Adequate levels of riboflavin can enhance swine productivity and health, contributing to more efficient production practices and potentially reducing environmental impacts.

## Data Availability

No datasets were generated or analysed during the current study.

## Abbreviations

AMP: Adenosine monophosphate
ATP: Adenosine triphosphate
CM: Control macrophages
COX-2: Cyclooxygenase-2
DLG: German Agricultural Society
EFSA: European Food Safety Authority
EGRAC: Erythrocyte glutathione reductase activity coefficients
FAD: Flavin adenine dinucleotide
FADT: Flavin adenine dinucleotide transporter
FAO: The Food and Agriculture Organization of the United Nations
FMN: Flavin mononucleotide
G-6P-D: Glucose-6-phosphate dehydrogenase
GSH: Reduced form of glutathione
GSH-Px: Selenium-dependent glutathione peroxidase
GSSG: Oxidized glutathione
HMGB1: High-mobility group box 1
iNOS: Nitric oxide synthase
MTHFR: Methylene tetrahydrofolate reductase
NAD+: Nicotinamide adenine dinucleotide
NADH: Dinucleotide nicotinamide molecules
NADPH: Nicotinamide adenine dinucleotide phosphate
NASEM: National Academies of Sciences, Engineering, and Medicine
NO: Nitric oxide
Nox2: NADPH oxidase 2
RBP: Riboflavin-binding protein
RF or RIBO: Riboflavin
RFK: Riboflavin kinase
RFVT: Riboflavin transporter
SAM: Seritoneal macrophages infected with Staphylococcus aureus
TNF: Tumor necrosis factor
TNFR1: Tumor necrosis factor receptor 1
